# The Effective Biodegradation of Poly(ε-caprolactone) by Engineered Yeast *Yarrowia lipolytica* Producing Lipase B

**DOI:** 10.3390/ijms27104625

**Published:** 2026-05-21

**Authors:** Żaneta Zdanowska, Lara Serrano-Aguirre, Aneta Krystyna Urbanek, Adam Dobrowolski, Aleksandra M. Mirończuk

**Affiliations:** 1 Laboratory for Biosustainability, Institute of Biology, Wrocław University of Environmental and Life Sciences, ul. Kożuchowska 5b, 51-630 Wroclaw, Poland; 130439@student.upwr.edu.pl (Ż.Z.); aneta.urbanek@upwr.edu.pl (A.K.U.); adam.dobrowolski@upwr.edu.pl (A.D.); 2 Polymer Biotechnology Group, Center for Biological Research Margarita Salas, Spanish National Research Council, 28006 Madrid, Spain

**Keywords:** lipase B, CALB, *Yarrowia lipolytica*, PCL, biodegradation

## Abstract

Poly(ε-caprolactone) (PCL) is a biodegradable aliphatic polyester with applications in many areas. Lipase B from *Moesziomyces antarcticus* (CALB, previously known as *Candida antarctica* lipase B) is a well-characterize enzyme capable of hydrolysing several polyesters. In this study, the codon-optimized gene encoding CALB was cloned into the yeast *Yarrowia lipolytica* to enhance its natural capabilities toward polyesters biodegradation. PCL films biodegradation was conducted directly in the medium using the engineered yeast at 28 °C. Process optimization employing baffled flasks significantly improved degradation efficiency and reduced time to 24 h. This study showed that the engineered yeast *Y. lipolytica* is a promising host for polyester biodegradation.

## 1. Introduction

Plastic has become an integral part of the modern world, but its persistence in the environment has turned it into a major source of pollution. According to recent data from Plastics Europe, global plastic production exceeded 430 million tons in 2024, with only a small fraction of the materials coming from recycling [1].This highlights the urgent need to look for new biotechnological strategies for plastic degradation, as conventional waste management methods have proven to be insufficient, energy-intensive, and ineffective.

Poly(ε-caprolactone) (PCL) is a biodegradable aliphatic polymer widely used in various industrial applications [2]. Its biodegradability stems from its aliphatic polyester backbone, which is more accessible to hydrolytic attack than other synthetic polymers. PCL is a thermoplastic material characterized by high flexibility and resistance to cracking, which makes it particularly valued for biomedical applications, including the production of scaffolds used in tissue engineering. In such context, its degradation in the body may range from several months to several years [3,4].

Lipase B from *Moesziomyces antarcticus* (CALB, previously known as *Candida antarctica* lipase B) is a versatile and extensively characterized biocatalyst widely applied in transesterification reactions in non-aqueous media, as well as in the hydrolysis of ester bonds under aqueous conditions. CALB is a monomeric protein consisting of 317 amino acids with a molecular mass of approximately 33 kDa [5]. It belongs to the α/β-hydrolase fold family, and its catalytic triad is composed of Ser105, Asp187, and His224. The nucleophilic serine residue forms part of the T-W-S-Q-G motif, which replaces the more common G-X_1_-S-X_2_-G motif found in most lipases. Moreover, CALB lacks the characteristic mobile lid that usually covers the active site entrance, thereby exposing the hydrophobic substrate-binding pocket directly to the solvent [6]. Its exceptional thermal stability and remarkable tolerance to organic solvents, together with its high enantioselectivity, make it suitable for a wide range of synthetic transformations, including kinetic resolutions, aminolysis, and esterification reactions [7].

From the perspective of polymer sustainability, it is necessary to consider not only biodegradability but also the potential for large-scale recycling and resource recovery. In the case of polyesters, CALB offers a unique advantage, as it catalyzes both the synthesis and the hydrolysis of ester bonds in aliphatic and aromatic polyesters. This dual functionality enables the enzymatic depolymerisation of polyesters into cyclic oligomers or monomeric products that can be further repolymerised or converted into value-added products [6]. The efficiency of this degradation process strongly depends on polymer physicochemical parameters such as crystallinity, glass transition temperature, chain mobility, and the presence of additives, which determine ester bond accessibility at the polymer surface.

In this context, CALB has been described to degrade various biodegradable polymers, including PLA, PBAT, PBS, and PCL, either through external addition (in soluble or immobilized form) or by embedding the enzyme into the polymer matrix [8,9,10,11,12]. Furthermore, CALB, together with the cutinase from *Humicola insolens* (HiC), has been reported to efficiently depolymerize PET, releasing terephthalic acid (TPA) [13]. Immobilized CALB has also been shown to hydrolyse bis(2-hydroxyethyl) terephthalate (BHET) into TPA and ethylene glycol (EG) or into mono(2-hydroxyethyl) terephthalate (MHET) and EG, depending on the reaction pH [14]. Thanks to its well-characterized structure, stability, and established immobilization protocols, CALB is among the most widely used enzymes in biocatalytic applications [15]. Its robustness and broad substrate specificity make it a valuable scaffold for the valorisation of mixed plastic streams through the combined depolymerization of waste polymers and synthesis of value-added products.

*Yarrowia lipolytica* is a non-conventional yeast that has found wide industrial application due to its suitability as a host organism. It belongs to the GRAS (Generally Recognized As Safe) group, meaning it can be safely used in the food, pharmaceutical, and biotechnology industries [16]. *Y. lipolytica* is capable of utilizing hydrophobic substrates such as fats, oils, and other lipophilic compounds. Moreover, *Y. lipolytica* is relatively easy to genetically modify, which facilitates the introduction and expression of heterologous genes from other microorganisms [17]. Taking advantage of these properties, this yeast has been used to address the environmental problem of plastic pollution.

The aim of this study was to optimize PCL biodegradation process by combining the natural capability of *Y. lipolytica* with the heterologous overexpression of CALB, hereafter referred to as LipCA.

## 2. Results

### 2.1. Cloning of LipCA Gene into Y. lipolytica and Its Functional Expression

**Figure 1:**
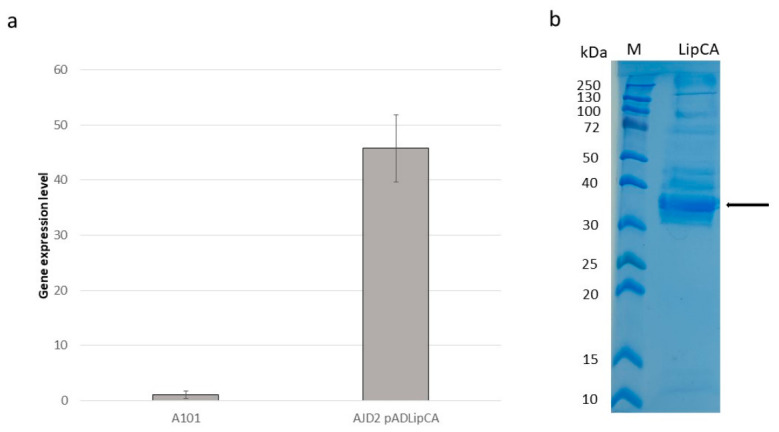
(**a**) Relative quantification of RNA transcript *LipCA* expression. Actin was used as a reference gene. Strains were grown in YPD medium. Samples were analyzed in triplicate and the standard errors were estimated using CFX96 Touch Real-Time PCR Detection System (Bio-Rad Laboratories, Inc., Hercules, CA, USA). (**b**). SDS-PAGE of the purified LipCA protein produced by *Y. lipolytica* AJD2 pAD-LipCA. As a marker WideRange Color Protein Ladder was used (A&A Biotechnology, Gdansk, Poland).

The first step in this study was the cloning of *LipCA* gene into *Y. lipolytica* AJD2 strain, which devoid of the two major extracellular proteases. To this end, a codon-optimized synthetic gene was fussed under UAS_B16_-TEF promoter [18] in the pAD vector [19], resulting in plasmid pAD-LipCA. The proper clone was then transformed into *Y. lipolytica*, the presence of the gene was confirmed and the gene expression was tested. A qRT-PCR analysis was done after 24 h of growth in YPD medium. The engineered strain, hereafter referred to as AJD2 pAD-LipCA, was compared to the wild-type strain A101 used as control. Total RNA was isolated and gene expression levels were tested. As shown in Figure 1a, high expression of *LipCA* was observed in the engineered strain. A residual expression was detected in the parental strain, most probably caused by non-specific amplification or cross-reactivity with one of the near twenty native lipase genes present in the *Yarrowia* genome [20].

To confirm the production of LipCA, an SDS-PAGE analysis was performed. The engineered strain AJD2 pAD-LipCA was grown in the YPD medium supplemented with 5% of glucose. After 96 h of cultivation, extracellular protein content was analyzed and overexpression of a protein with a molecular weight of around 37.1 kDa was observed, corresponding to the LipCA (Figure 1b).

### 2.2. Cell’s Conditions and Capability of Growth

**Figure 2:**
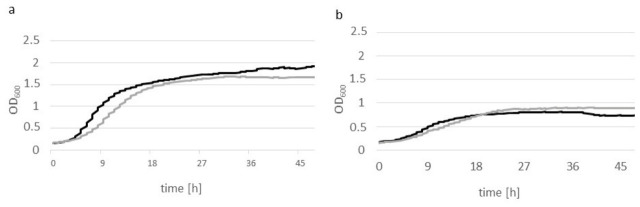
Growth of *Y. lipolytica* A101 wild-type strain (black) and *Y. lipolytica* AJD2 pAD-LipCA (gray) on YPD medium with 5% (*w*/*v*) glucose (**a**) and YNB medium with 5% (*w*/*v*) glucose (**b**).

The production of the synthetic gene may impose a metabolic burden on the host cell, as the use of a strong hybrid promoter for heterologous protein expression may lead to the depletion of transcriptional factors. To assess the physiological state of the cells, the growth of the strains A101 and AJD2 pAD-LipCA was monitored using a Tecan microplate reader in YPD and YNB medium both supplemented with 5% (*w*/*v*) glucose. As shown in Figure 2, both strains exhibited similar growth dynamics, with minor differences observed between minimal (a) and rich (b) medium. In YNB medium, both strains showed moderate growth, reaching OD_600_ below 0.9. On the contrary, both strains showed robust growth in YPD medium, with OD_600_ exceeding 1.5 after 18 h of cultivation. These results indicate that the engineered strain maintains high viability and that overexpression of the heterologous protein does not significantly impair biomass production.

### 2.3. Biodegradation of PCL—Spot Test and Shake-Flask Cultures

**Figure 3:**
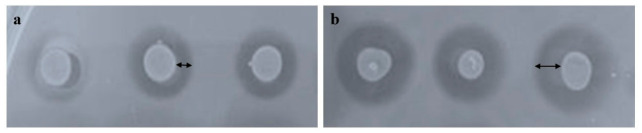
Spot test on 0.1% (*w*/*v*) PCL plates after incubating *Y. lipolytica* AJD2 (**a**) and AJD2 pAD-LipCA (**b**) strains for 24 h.

The qRT-PCR experiment and SDS-PAGE confirmed that *LipCA* gene is properly expressed in the AJD2 strain and LipCA protein is produced. Therefore, the next step in our research was to verify the enzymatic activity of the engineered strain toward PCL. To this end, overnight (ON) cultures of the partental strain AJD2 (the control) and the strain harboring the overxpression cassette, AJD2 pAD-LipCA, were spotted onto YNB medium supplemented with 0.1% (*w*/*v*) PCL emulsion. As shown in Figure 3, both strains showed activity toward PCL after 24 h of growth. This effect is attributed to the natural production of lipases by *Y. lipolytica* [21], however it was clearly enhanced by the secretion of LipCA (Figure 3b). The halo size increased over time, and after 72 h the whole plate became transparent). The engineered strain exhibited higher activity, most likely due to synergic activity of the native lipases and the hetereologus LipCA.

**Figure 4:**
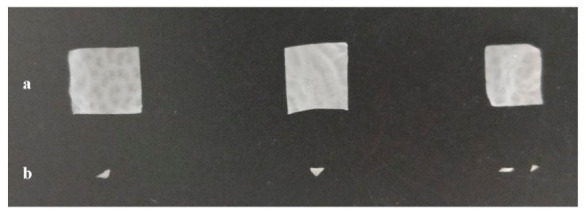
PCL films biodegradation by *Y. lipolytica* wild-type strain A101 (**a**) and engineered AJD2 pAD-LipCA strain (**b**) after 96 h at 28 °C of shaken cultivation in YPD medium (5% glucose) in Erlenmeyer flasks.

To evaluate the biodegradation capability of the engineered *Y. lipolytica* strain overexpressing LipCA, the shake-flask experiments were performed using PCL films (approximetly 0.1 g) in YNB medium supplemented with 5% (*w*/*v*) glucose. The engineered AJD2 pAD-LipCA strain was compared to the A101 wild-type strain. The high concentration of glucose did not impair yeast growth, as *Y. lipolytica* naturally produces erythritol which acts as an osmoprotectant [22]. Minimal medium was initially selected to assess whether environmental conditions would be succifient for biodegradation process. The tested strains were grown at 28 °C with vigorous shaking (200 rpm) and biodegradation was monitored every 24 h. After 96 h of incubation, visible degradation of PCL films was observed in the engineered strains cultures (Figure 4). In contrast, films incubated with the wild-type strain (three independent biological replicates) remained largely intact. These results demonstrate that overexpression of LipCA significantly improve PCL biodegradation.

**Figure 5:**
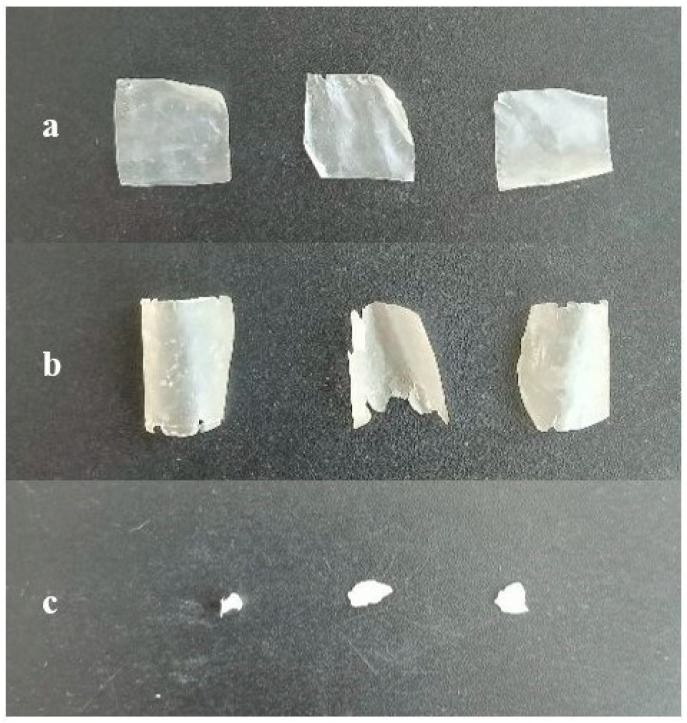
PCL films recovered after 24 h of shaken flask cultures at 28 °C in YPD medium supplemented with 5% (*w*/*v*) glucose, in baffled flasks containing: the medium without yeast (**a**), the *Y. lipolytica* wild-type strain A101 (**b**) and the engineered strain AJD2 pAD-LipCA (**c**).

*Y. lipolytica* is a yeast with high demand for dissolved oxygen (DO) in the culture medium [23]. Oxygen limitation has been shown to negatively affect its physiological state, making this parameter critical for industrial applications [19]. To further optimize the PCL biodegradation process, baffled flasks were used to increase DO levels and YPD medium was selected to enhance cell growth and, consequently, protein production. As a negative control, PCL films were incubated in the medium without any yeast strain to verify that mechanical forces during shaking did not contribute to film deterioration. All strains were grown under the same conditions. Process optimization significantly improved effectiveness of PCL biodegradation. After 24 h of cultivation, visible residues of PCL films were observed in the cultures of the engineered strain (Figure 5 and Figure S1). Control experiments confirmed that mechanical forces alone did not impair the integrity of the films (Figure 5a). However, the use of YPD medium and increased DO levels also improved the wild-type strain capabilities toward PCL biodegradation (Figure 5b). In the cultures of the engineered strain (Figure 5c), film fragments were difficult to recover due to their reduced size. To further investigate surface changes in the films, selected samples were analyzed by scanning electron microscopy (SEM).

### 2.4. Scanning Electron Microscopy (SEM) Analysis

**Figure 6:**
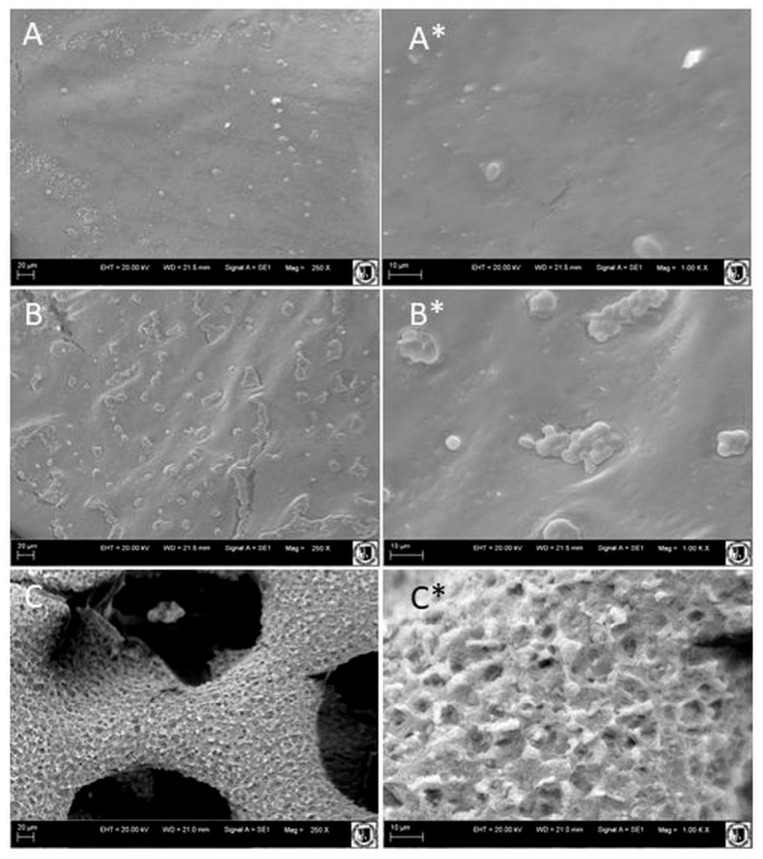
SEM images of PCL films recovered from shake-flask cultures after 24 h of incubation at 28 °C; (**A**) control PCL film in YPD medium, (**B**) wild-type A101 strain; (**C**) engineered AJD2 pAD-LipCA strain. **Left Panels**: scale bar 20 um, EHT = 20.00 kV; WD = 21.5 mm; Signal A = SE1; Mag = 250×, Logo of UWPr; **Right Panels**: Scale bar: 10 um, EHT = 20.00 kV; WD = 21.5 mm; Signal A = SE1; Mag = 1.00 k×, Logo of UWPr regards.

As mentioned above, samples of PCL films incubated in medium alone, medium with the wild-type strain, and medium with the engineered strain were taken for SEM analysis. The PCL films degraded over time are presented in Figure 6. The films incubated without any microbial activity exhibited smooth surfaces, without any visible damages (Figure 6A). In contrast, films incubated with A101 strain became rough with small torn areas and yeast cells attached to the surface (Figure 6B*). This confirmed that *Y. lipolytica* possesses an inherent capacity for polymer biodegradation, as the initial step in plastic biodegradation involves cell adhesion to the hydrophobic surface [24]. The most pronounced surface alterations were observed in films incubated with the strain overexpressing LipCA (Figure 6C). These samples exhibited greater roughness of the surface and the presence of deep cracks and rounded holes. The extent of the surface damages was consistent with the macroscopic observations (Figure 5c). Overall, these results indicate that PCL degradation by the yeast *Y. lipolytica* is significantly enhanced by the heterologous overexpression of the LipCA. This lipase activity likely promotes the formation of big pits and cracks in the surface of PCL films, thereby accelerating the biodegradation process.

## 3. Discussion

Biodegradable polymers have emerged as a promising alternative to conventional plastics derived from fossil fuels, primarily due to their enhanced susceptibility to biological degradation. Among them, poly(ε-caprolactone) (PCL), a petroleum-based aliphatic polyester, has been widely studied as a model substrate. However, its reported degradation rates vary significantly across studies, making it difficult to establish a consistent and reliable timeframe for biodegradation [2]. This variability reflects not only differences in experimental conditions but also the diversity of biological systems and enzymes involved.

The degradation of aliphatic polyesters such as PCL is often considered a useful model for addressing more recalcitrant polymers, including aromatic polyesters. Nevertheless, enzyme specificity remains a critical factor, as certain hydrolases exhibit distinct preferences for aliphatic or aromatic substrates [25]. Therefore, identifying and optimizing efficient enzymatic systems is essential for advancing polymer biodegradation strategies.

Most studies to date have relied on purified enzymes, which, although effective, require considerable time, cost, and processing steps that may limit their scalability. In contrast, whole-cell biocatalytic systems offer a more practical and economically viable alternative. In our previous work, we demonstrated that direct biodegradation of PCL using *Y. lipolytica* cultures represents a simple and low-cost approach [17]. Building on this concept, the present study introduces a significant improvement through the overexpression of LipCA from *M. antarcticus*, in the yeast *Y. lipolytica*. This yeast is particularly well suited for such applications due to its natural ability to secrete hydrolases. The combined, potentially synergistic activity of endogenous enzymes and the heterologous LipCA appears to play a key role in enhancing degradation efficiency. Importantly, this strategy led to a substantial reduction in degradation time, from 144 h to 24 h, highlighting the effectiveness of the engineered system. This result also improves previous results from the literature on the purified enzyme, where the biodegradation process took more than 64 h [12]. Beyond the shorter processing time, the use of a whole-cell system eliminates the need for enzyme purification and may facilitate continuous or large-scale applications.

Overall, these findings support the potential of engineered *Y. lipolytica* as a robust platform for polyester biodegradation and provide a foundation for further optimization and extension toward more complex polymer systems.

## 4. Materials and Methods

### 4.1. Strains and Media

The following strains of *Yarrowia lipolytica* were used in this study: the wild-type strain A101, the parental strain AJD2 [26], and the engineered strain AJD2 pAD-LipCA. The strains were cultivated in two media: YNB minimal medium (Sigma-Aldrich, Darmstadt, Germany) containing 5% (*w*/*v*) glucose and YPD medium (A&A Biotechnology, Poland) with 5% (*w*/*v*) glucose. For RNA isolation, the strains were pre-cultured in YPD medium. Shaking flask experiments to test lipase activity were performed in Erlenmeyer flasks containing YNB medium (with 5% glucose) at 28 °C and 200 rpm. Additional experiments were carried out in baffled flasks containing YPD medium (with 5% glucose) under the same conditions (28 °C, 200 rpm). Spot tests were performed on YNB medium supplemented with 0.1% (*w*/*v*) poly(ε-caprolactone) (PCL). The PCL film was prepared by dissolving 0.03 g of the polymer in 1 mL of dichloromethane. A thin layer of the solution was poured onto a Petri dish and left to dry for 5 h. After drying, the PCL film was cut into 1 cm × 1 cm squares and sterilized with 70% (*v*/*v*) ethanol [27].

### 4.2. Cloning and Transformation Protocols

All restriction enzymes were purchased from FastDigest Thermo Scientific^TM^ (Waltham, MA, USA) and all of the digestions were performed according to standard protocols. The PCR reactions were set up using recommended conditions and Phusion high-fidelity DNA polymerase (Thermo Scientific^TM^). The ligation reactions were performed for 15 min at room temperature using T4 DNA Ligase (Thermo Scientific^TM^). The *E. coli* minipreps were performed using the Plasmid Mini Kit (A&A Biotechnology, Gdansk, Poland). Transformation of *E. coli* strains was performed using standard chemical protocols). Genomic DNA (gDNA) was extracted from *Y. lipolytica* using the Genomic Mini AX Yeast Spin kit (A&A Biotechnology, Gdansk, Poland). The obtained plasmid was digested with *MssI* to create linear expression cassettes devoid of *E. coli* DNA and surrounded by *Y. lipolytica* rDNA for targeted integrations. The transformants were plated out on selective media and were confirmed via gDNA extraction and three distinct PCR confirmations [28].

### 4.3. Strain Construction

First, LipCA from *Candida antarctica* (https://www.uniprot.org/uniprotkb/P41365/entry (accessed on 4 April 2025)) was codon-optimized (1086 bp) for *Y. lipolytica* and fused to the XPR2 signal sequence from this yeast for protein secretion. Gene was cloned into the pAD vector using *SgSI* and *NheI* sites, resulting in pAD-LipCA vector. The proper construct containing overexpression cassette was sequenced (Genomed, Warszawa, Poland). The newly obtained vectors were digested with *MssI*, resulting in linear expression cassettes with *Y. lipolytica* rDNA, for integration in the *Y. lipolytica* genome. *Y. lipolytica* transformation was described previously [29]. The proper gene expression was confirmed by RNA isolation and qRT-PCR.

### 4.4. RNA Isolation and qRT-PCR

The cultures for RNA isolation were grown in 10 mL of YPD medium in 100 mL flasks at 28 °C and 200 rpm. Samples were collected after 24 h of incubation. The RNA isolation and qRT-PCR procedure were as described before [30]. Primers q*LipCA*-F (5′-CTGGCCAAGCTCTTGTCTCTG-3′) and q*LipCA*-R (5′-GTGAGTCCTGCATCAA GAAC-3′) resulted in the product size of 140 bp. The results were normalized to the actin gene amplified with the primers qACT-F (5ʹ-GAGTCACCGGTATCGTTC-3′) and qACT-R (5′-GCGGAGTTGGTGAAAGAG-3′). Samples were analyzed in triplicate in CFX Connect and the data analysis was carried out in CFX Maestro Software 2017 (Bio-Rad, USA).

### 4.5. Protein Production and Purification

Overnight culture of *Y. lipolytica* AJD2 pAD-LipCA was used to inoculate 50 mL of YPD medium supplemented with 5% glucose. After 96 h of cultivation, the cells were spun down and supernatant was used for follow procedure. Ammonium sulfate (AS) was added to the supernatant in 60% saturation at 4 °C with stirring. The mixtures were centrifuged at 14,000× *g* at 4 °C for 20 min. Obtained pellet was dissolved in 1 mL of 50 mM HEPES. The molecular mass of the protein was confirmed by SDS-PAGE using 15% polyacrylamide gel. WideRange Color Protein Ladder (A&ABiotechnology, Gdańsk, Poland) was used as a molecular weight marker.

### 4.6. Growth Curves

The growth characteristics of the strains were tested in 96-well plates using a Spark reader (Tecan Group Ltd., Männedorf, Switzerland). A standardized cell suspension (OD_600_ = 0.1) was incubated in 200 µL of YPD medium or YNB medium (2% glucose) at 28 °C for 48 h with shaking. Optical density (OD_600_) measurements were performed automatically every 30 min. The experiment was performed in three independent biological replicates.

### 4.7. Spot Test

Overnight (ON) cultures of the tested strains were incubated in YPD medium, optical density (OD_600_) was adjusted to 0.1 and 2 µL of the cultures were plated onto YNB medium with 0.1% (*w*/*v*) emulsified PCL as described before [27].The plates were incubated at 28 °C and after 24 h the development of clear zones was observed. The test was performed to determine whether the introduced *LipCA* lipase gene was functional and whether it could effectively degrade polyester. The clone with the highest activity (the widest halo) was chosen for further experiments.

### 4.8. Shaking Flasks Culture

The experiments were carried out using the prepared PCL film cut into 1 × 1 cm squares and sterilized by rinsing in 70% ethanol and UV irradiation (10 min). The cultures were grown in 250 mL Erlenmeyer or baffled flasks containing 50 mL of YPD medium supplemented with 5% of glucose [24]. The inoculum was a 72 h preliminary culture, and the initial OD in the experimental flasks was set at 0.5. As negative control, flasks with medium and film, without the addition of yeast, were used. Apart from the engineered strain, the wild-type strain *Y. lipolytica* A101 was also used in this experiment. The cultures were incubated at 28 °C (200 rpm) for 96 or 24 h for Erlenmeyer or baffled flasks, respectively.

### 4.9. Scanning Electron Microscopy (SEM)

In order to verify the influence of the microorganism’s activity on the polymer plastic surface, a scanning electron microscope was used. The investigation was conducted on three biological replicates for each strain bearing overexpressing cassette, as a control medium with PCL films incubated under the same conditions, was used. Film samples were mounted on standard SEM stubs using conductive carbon adhesive tape. The sample surfaces were subsequently sputter-coated with a thin gold (Au) layer for 300 s, resulting in a coating thickness of up to 2 nm. The sputtering process was carried out using an Edwards Scancoat Six Sputter Coater (Burgess Hill, UK) to enhance surface conductivity and minimize charging effects during electron beam exposure. Microstructural and surface topography analyses were performed using a ZEISS EVO LS15 (ZEISS, Oberkochen, Germany) operating at an accelerating voltage (EHT) of 20 kV. Imaging was conducted in high-vacuum mode using a secondary electron detector (SE1) to obtain detailed surface morphology and dedicated software. All SEM observations were carried out at the Laboratory of Electron Microscopy, Wrocław University of Environmental and Life Sciences, Poland.

## 5. Conclusions

In this study, the engineered *Y. lipolytica* AJD2 pAD-LipCA strain was successfully applied for the degradation of the aliphatic polyester PCL. The effect of growth conditions was evaluated by comparing two types of medium and flask configurations. The results demonstrated that PCL degradation was significantly accelerated in cultures grown in YPD medium using baffled flasks. Importantly, a substantial improvement in the degradation of PCL films by the engineered *Y. lipolytica* strain was achieved though simple optimization of cultivation conditions, without additional genetic modifications. Given the high oxygen demand of *Y. lipolytica*, the use of baffled flasks increases aeration, thereby supporting improved cell growth and enzymatic activity. Increasing dissolved oxygen (DO) levels proved to be a key factor in enhancing process efficiency.

Overall, these findings highlight that process optimization, particularly through improved oxygen transfer, can significantly enhance biodegradation performance while contributing to a more efficient and cost-effective system.

## Data Availability

The original contributions presented in this study are included in the article/Supplementary Material. Further inquiries can be directed to the corresponding author.

## Supplementary Materials

The following supporting information can be downloaded at: https://www.mdpi.com/article/10.3390/ijms27104625/s1.
