# Supplementary material for: The Effective Biodegradation of Poly(ε-caprolactone) by Engineered Yeast Yarrowia lipolytica Producing Lipase B
Source: Int J Mol Sci. 2026 May 21;27(10):4625. doi: 10.3390/ijms27104625 (PMC13207279; doi:10.3390/ijms27104625)
Supplement: Supplementary file 1 [file ijms-27-04625-s001.zip › ijms-4314406-supplementary.pdf]

International Journal of *Molecular Sciences*

MDPI

Supplementary Material

The effective biodegradation of poly(ε-caprolactone) by engineered yeast *Yarrowia lipolytica* producing Lipase B

Żaneta Zdanowska[1†], Lara Serrano-Aguirre[1,2,±†], Aneta K. Urbanek[1], Adam Dobrowolski[1] and Aleksandra M. Mirończuk[1*]

[1] Laboratory for Biosustainability, Institute of Biology, Wrocław University of Environmental and Life Sciences, ul. Kożuchowska 5b, 51-630, Wroclaw, Poland,

[2] Polymer Biotechnology Group, Center for Biological Research Margarita Salas, Spanish National Research Council, Madrid, Spain

† These authors contributed equally to this work

± Current address: Entzimatiko, Zumaia, Spain

*corresponding author aleksandra.mironczuk@upwr.edu.pl

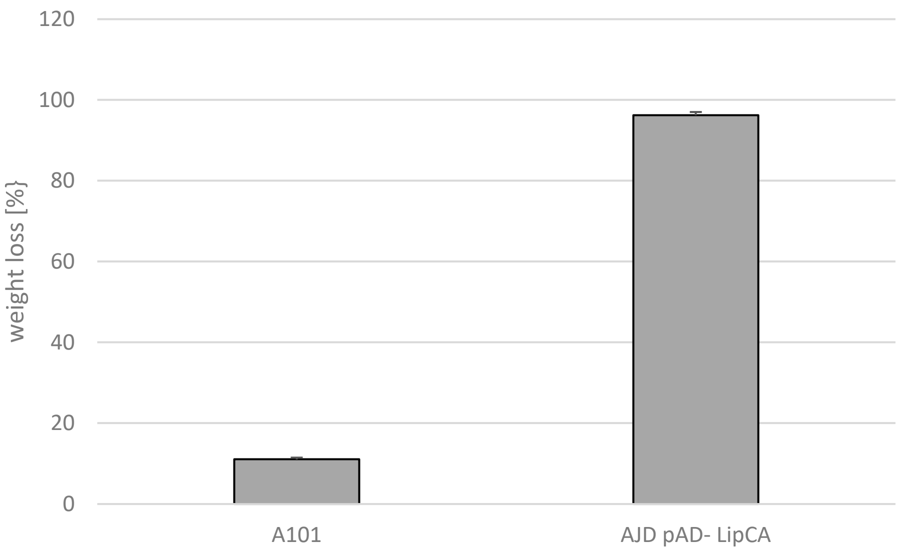

Figure S1. Weight loss of the PCL films incubated in the baffled shake-flasks after 24 hours. Cultivation was performed at 28 °C at constant agitation (200 RPM) in YPD medium. Three biological replication was used in this experiment. Error bars present SD.
